# Automated atomic absorption determination of lead in gasoline

**DOI:** 10.1155/S1463924682000297

**Published:** 1982

**Authors:** J. H. Lowry, T. J. Meszaros, L. Conlon

**Affiliations:** Environmental Protection Agency National Enforcement Investigations Center Building 53, Box 2.5227 Denver Federal Center Denver Colorado 80225 USA


Journal of Automatic Chemistry, Volume 4, Number 3 (July–September 1982), pages 112–115

### Automated atomic absorption determination of lead in gasoline

**J. H. Lowry, T. J. Meszaros and L. Conlon**

*Environmental Protection Agency, National Enforcement Investigations Center, Building 53, Box 25227, Denver Federal Center, Denver, Colorado 80225, USA*

### Introduction

The determination of lead in gasoline by atomic absorption spectrometry has been adopted by the American Society for Testing Materials [1] and the Environmental Protection Agency (EPA) [2] as the standard method of analysis. The method consists of the manual preparation of an *in situ* reaction of the alkyl lead compounds in gasoline with iodine, stabilization of the alkyl lead iodide complexes with tricapryl methyl ammonium chloride (Aliquot 336), 10-fold dilution with methyl isobutyl ketone (MIBK) and measurement by atomic absorption spectrometry with an air-acetylene flame. The iodine reaction eliminates the problem of variations in response due to different alkyl lead compounds, Kashiki *et al.* [3]. The dilution compensates for severe non-atomic absorption, scatter from unburned carbon, and minimizes matrix effects, see Lukasiewiez *et al.* [4].

The EPA has initiated an annual, nationwide survey to determine the extent of tampering with the pollution-control device fitted on American automobiles. Another purpose of these surveys is to determine if fuel switching has occurred, i.e. whether leaded gasoline is being used in automobiles designed for use of unleaded gasoline (< 0·05 g Pb/gal.). Consequently, these surveys require the analysis of numerous gasoline samples' lead content. This demand necessitates accurate, rapid analyses by use of an automated method.

Heistand *et al.* [5] automated a nitric acid extraction atomic absorption method for the analysis of lead in gasoline. They stated that the standard method could not be automated because the pump tubing deteriorates rapidly in the presence of MIBK. The data generated by the EPA must be legally defensible and must be comparable with data gathered by the manual standard method. Hence, Heistand *et al.*'s automated method was deemed inappropriate for the authors' application.

The automation of the standard method is discussed below. The incompatibility of the MIBK with the pump tubing was initially circumvented by the use of solvent displacement flasks and, later, by use of constant-flow syringe pumps. Data showing equivalence of the automated and manual procedures, and precision and accuracy data, gathered over a four-month period during the analysis of about 1500 samples are presented here. The effects of holding times, container types and storage conditions on the lead content of gasoline samples are also discussed. The findings indicate a definite need to specify these as requisites in the standard method.

### Experimental

### *Apparatus*

A Technicon Autoanalyzer Sampler and a Pump III were used for the automated system. Standard heating block coils (No. 157-0225) were used for mixing coils because of the need for good mixing with the high flow-rate. A Perkin-Elmer Model 403 atomic absorption spectrophotometer and a strip-chart recorder were the detection system used for the manual and automated procedures.

### *Reagents*

Working standards of lead alkyls in reference fuel (US Environmental Protection Agency, Research Triangle Park, North Carolina, USA) were utilized in both procedures. For the manual procedures the iodine solution (Fisher Scientific Company, Fairlawn, New Jersey, USA) was 3% w/v in toluene (Burdick & Jackson, Muskegon, Michigan, USA). The automated procedure iodine solution concentration was 0·24% w/v in toluene. In both procedures the Aliquot 336 (Aldrich Chemical Company, Milwaukee, Wisconsin, USA) solution was 0·88% in MIBK (Burdick & Jackson). Certified unleaded gasoline was obtained from the Phillips Chemical Company, Borger, Texas, USA.

### Procedure

### *Manual*

The procedure published in the Federal Register [2] was followed, the only exception being that alkyl lead compounds in isoctane standards were used instead of lead chloride standards.

### *Automated*

The flow diagram of the automated system is shown in figure 1. A sampling rate of 30 samples/h with a 2 : 1 sample-to-wash ratio provides sufficient peak resolution to establish a base-line at concentrations less than 0·05 g Pb/gal. The procedure screened all samples at a 30/h sampling rate. Any samples with a lead content greater than 0·05 g/gal. were rerun at a sampling rate of 20/h with a 3 : 1 sample-to-wash ratio. The wash solution was certified as unleaded gasoline. The sample was diluted and mixed in the first mixing coil with MIBK displaced from a 2 l flask with distilled water. The iodine reagent (0·24% w/v) was then reacted with the air segmented stream in the second mixing coil. At the flow rates given in figure 1, the reaction time of the iodine before the addition of the Aliquot 336 was a little over 1 min. The Aliquot 336 solution was introduced into the system by means of displacement from a 500 ml flask again using distilled water. The air segmented stream was then debubbled by reverse displacement and the products of the reaction were pumped into the atomic absorption spectrophotometer. The operating conditions of the atomic absorption spectrophotometer were as follows: wavelength: 283·3 nm; acetylene flow: 20 ml/min.; airflow: 65 ml/min.; nebulizer flow: 5·2 ml/min.

 0142-0453/82/0403-0112 $05.00 © 1982 Taylor & Francis Ltd

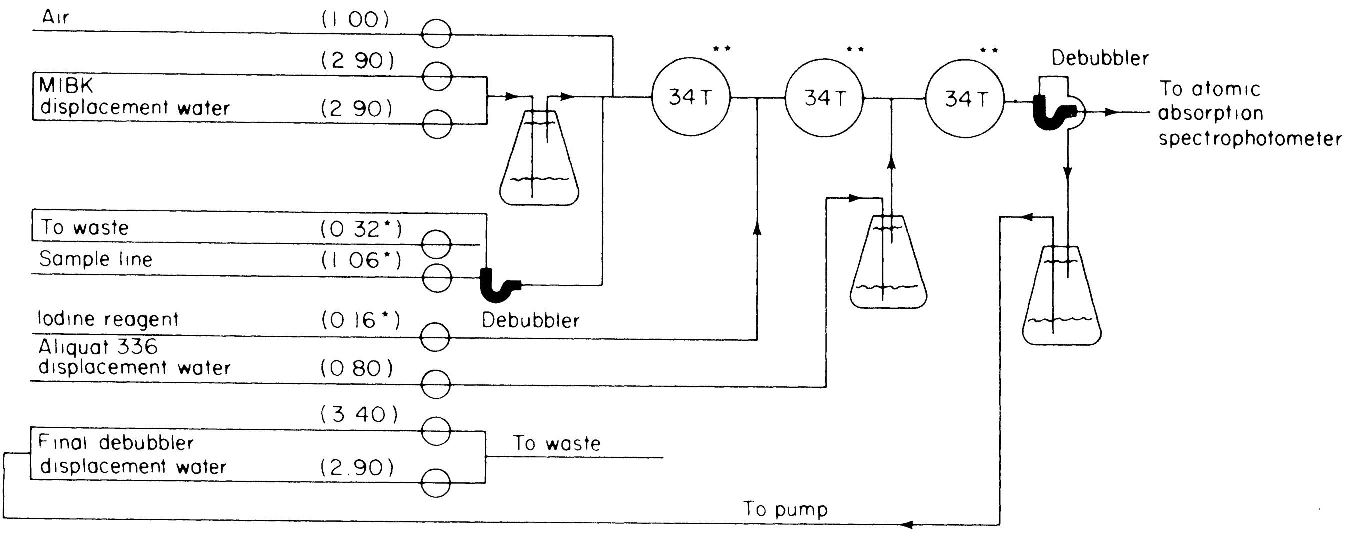

*Figure 1. Flow diagram for the automated system. Where * is Solvaflex pump tubing and ** is Technicon part No. 157-0225.*

The solvent displacement flasks are Erlenmeyer flasks fitted with silicone rubber stoppers and glass tubing. Later, Model 220 Sage constant-flow syringe pumps fitted with 20 ml Teflon-coated syringes were used for the addition of MIBK. The solvents are transported through Teflon tubing fitted to the glass with heat-shrinkable Teflon tubing. The glassware used in the manifold is interconnected with polyethylene tubing because tygon tubing dissolves in the presence of MIBK. Solvaflex tubing was compatible with the gasoline samples. However, the iodine reagent solvaflex pump tubing had to be changed daily.

### *Sample storage study*

Containers made of polyethylene, tin with soldered seams, and tin with pressed seams were used in a sample storage study. In addition, tin containers with pressed seams were used with and without a tin-cap insert.

Seven aliquots of the composite sample were analysed to determine the zero day lead content. Forty-eight 50 ml aliquots were transferred on the same day to the individual sample containers. At intervals of one, two, four and 23 weeks, three samples of each container type stored at 4°C or at ambient temperature (six samples/container type/interval) were allowed to equilibrate at room temperature and then analysed with the automated system. Subsequently, a similar study was performed with glass containers using a 2 gal. composite test sample.

### Results and discussion

### *Manual and automated comparison*

The additions of the individual reagents of the automated sysem were designed to match as closely as possible those of the manual method. Table 1 shows the volume of each reagent required by the manual method to the volumes of reagents utilized in 1 min. by the automated method. Iodine at a concentration of 3% w/v caused the pump tubing to harden very quickly. The iodine was diluted and its pump flow rate was increased to result in an equal molar concentration addition. The percentage total volume and molar concentration of each reagent is very closely matched in both methods.

The comparability between two methods of analysis is usually measured by defining the sensitivity, precision, and accuracy of each method. Four-point calibration curves were prepared over the concentration range of 0·010 g/gal. to 0·110 g/gal. for both methods. A least squares fit of the calibration data for the manual and automated methods resulted in slopes of 9·8 and 9·1 Abs. Units/g Pb/gal. and intercepts of −0·016 and 0·012 respectively. This demonstrates that both systems show similar sensitivity.

The percentage relative standard deviation (% RSD) of replicate analysis of a sample is a measure of the precision of the method. Table 2 shows the results of the replicate analysis of three samples by the automated method and the replicate analysis of one sample by the manual method. A comparison of the % RSD of the respective methods indicates that the precision is very similar. Another measure of precision can be obtained by the absolute difference of duplicate analysis. Four samples analysed in duplicate by both methods resulted in the data presented in table 2. The average difference for duplicate analysis by both methods is less than 0·005 g/gal., which is the maximum acceptable difference allowed by the standard method. In consideration of these measures of precision, the precisions of both methods are approximately equal.

The accuracy of both methods was assessed by measuring the lead content of three levels of National Bureau of Standards (NBS) certified gasoline standards. The results of these analyses are given in table 2. The manual analysis average deviation was biased low, −1·3% ± 5·7%, while the automated analyses are biased high, 2·0% ± 3·7%. Absolute deviations would indicate that the two methods are comparable.

Seventeen unleaded gasoline samples were analysed by both methods. The results of these analyses are presented in table 3. A statistical student T method comparison test indicates there is no statistical difference between the results of the two methods.

There are a number of practical considerations that favour the use of the automated analysis in a routine laboratory situation. When a work-load of 20 samples is on hand, the automated analysis results in substantial savings in the cost of labour and reagents over manual analysis. In a one-man day, at least 100 sample analyses can be performed by the automated analysis procedure. If account is taken to the time involved in cleaning glassware, in sample preparation as well as analysis, then only about 25 sample analyses can be performed manually. The ease with which quality-control data can be gathered with the automated analysis offers a significant advantage; these data are important for legally defensible analyses. An additional advantage of the automated system is that since the entire system is closed, MIBK vapours are substantially minimized in the laboratory.

*Table 1. Comparison of reagent usage by the manual and automated methods.*

| Reagent proportions | | | | |
|---|---|---|---|---|
| Manual | | | Automated | |
| ml used in 1 min. | % total volume | Reagent | ml used in 1 min. | % total volume |
| 39·9 | 79·8 | MIBK | 5·80 | 77·3 |
| 5·0 | 10·0 | Standard or sample | 0·74 | 9·9 |
| 0·1 | 0·2 | $I_2$/toluene | 0·16 | 2·1† |
| 5·0 | 10·0 | 1% Aliquot 336/MIBK | 0·80 | 10·7 |
| 50·0 | 100·0 | Total volume | 7·50 | 100·0 |

†Concentration of $I_2$/toluene solutions are 3% for manual method and 0·24% for automated method.

*Table 2. Precision and accuracy data for the manual and automated methods.*

Precision

*Replicate analysis*

| Number of analyses | Method | Concentration in g Pb/gal. | %RSD |
|---|---|---|---|
| 5 | Manual | 0·054 | 3·6 |
| 5 | Automated | 0·010 | 4·2 |
| 5 | Automated | 0·048 | 3·5 |
| 5 | Automated | 0·085 | 3·3 |

*Duplicate analysis*

| Sample number | Automated Average | Automated Difference | Manual Average | Manual Difference |
|---|---|---|---|---|
| 1 | 0·031 | 0·003 | 0·033 | 0·001 |
| 2 | 0·043 | 0·000 | 0·045 | 0·002 |
| 3 | 0·012 | 0·004 | 0·015 | 0·001 |
| 4 | 0·101 | 0·003 | 0·098 | 0·008 |
| | $\bar{x}_d = 0·0025$ | | $\bar{x}_d = 0·0030$ | |
| | SD = 0·0017 | | SD = 0·0034 | |

Accuracy

NBS reference standards

Manual

| $N$ | NBS value (g Pb/gal.) | $\bar{x}_{deviation}$ | $\sigma_{deviation}$ |
|---|---|---|---|
| 8 | 0·0322 | −2·1 | 6·2 |
| 20 | 0·0519 | 0·5 | 4·6 |
| 20 | 0·0725 | −2·4 | 6·2 |

Automated

| $N$ | NBS value (g Pb/gal.) | $\bar{x}_{deviation}$ | $\sigma_{deviation}$ |
|---|---|---|---|
| 5 | 0·0322 | −2·7 | 1·7 |
| 18 | 0·0519 | 1·6 | 5·7 |
| 7 | 0·0725 | 1·6 | 3·8 |

*Table 3. Statistical comparison of actual sample analyses by the manual and automated methods.*

| Sample number | Found value g/gal. Manual | Found value g/gal. Automated | Difference between methods |
|---|---|---|---|
| 1 | 0·032 | 0·032 | 0·000 |
| 2 | 0·044 | 0·043 | −0·001 |
| 3 | 0·014 | 0·014 | 0·000 |
| 4 | 0·094 | 0·099 | +0·005 |
| 5 | 0·035 | 0·034 | −0·001 |
| 6 | 0·053 | 0·052 | −0·001 |
| 7 | 0·012 | 0·007 | −0·005 |
| 8 | 0·032 | 0·032 | 0·000 |
| 9 | 0·014 | 0·010 | −0·004 |
| 10 | 0·086 | 0·092 | +0·006 |
| 11 | 0·055 | 0·058 | +0·003 |
| 12 | 0·032 | 0·030 | −0·002 |
| 13 | 0·012 | 0·007 | −0·005 |
| 14 | 0·050 | 0·055 | +0·005 |
| 15 | 0·074 | 0·073 | −0·001 |
| 16 | 0·010 | 0·005 | −0·005 |
| 17 | 0·089 | 0·097 | +0·008 |

where $\bar{x}_{difference} = 0·000\,12$, $\delta_{difference} = 0·004$, $N = 17$,

$t = \frac{\bar{x}}{\delta}\sqrt{\eta} = 0·123$. For $N - 1 = 16, t_{05} = 1·746$.

### Survey sample analysis

The in-house use of the automated procedure places a heavy emphasis on quality control. This procedure first requires a check of the slope of the calibration curve. All calibration curves used for sample analyses agreed to within 10% of the slope earlier. Every 10th sample was analysed in duplicate. At least one NBS reference standard and at least one blind Research Triangle Park reference standard were analysed during an analysis run. All samples with a peak height greater than the 0·05 g/gal. standard were rerun and spiked with known quantities of alkyl lead compounds. Samples were diluted, if necessary, with unleaded gasoline so that the resulting diluted value, as well as the diluted spike sample valve, occurred in the calibration curve range.

The quality-control results of the analysis of 364 samples of the 1491 total analysed are summarized in table 4. The average difference of the duplicate analyses very closely approximates to zero, which would be expected statistically. Within this 95% confidence interval, all duplicate analyses performed with the automated system would differ by less than 0·0046 g/gal., which is within the acceptable limit of 0·005 g/gal. difference established in the standard method. The accuracy of the method evaluated over an extensive period is quite good. Within a 95% confidence interval, values reported over 0·05 g/gal. are within 10% of the true value. Values reported below 0·05 g/gal. are within 15% of the true value. Data obtained by spiking samples indicate that no substantial matrix effects were encountered in the analyses.

The percentage of unleaded gasoline-designed automobiles that switched to leaded gasoline are summarized by State in table 5; the data are from an *Eight State Survey* carried out in 1979. The average fuel switching percentage was 9·3%. The difference between the Vermont I and II studies shown in the table is that the Vermont II study population included a higher percentage of automobiles owned by people living in rural areas.

*Table 4. Precision and accuracy data for the automated method gathered during the EPA survey (total samples analysed = 1491).*

| *N* | Precision duplicate analyses (average difference [g Pb/gal.] |  | Standard deviation (SD) |
|---|---|---|---|
| 156 | 0·00011) |  | 0·0023 |
|  | Accuracy NBS reference standards |  |  |
| *N* | Concentration (g Pb/gal.) | Average % deviation | SD |
| 21 | 0·0322 | 3·4 | 6·4 |
| 36 | 0·0519 | 0·7 | 4·8 |
| 20 | 0·0725 | 0·7 | 4·9 |
|  | 'Blind' reference standards |  |  |
| *N* | Average difference |  | SD |
| 23 | −0·0009 |  | 0·004 |
|  | Spiked samples |  |  |
| *N* | Average % recovery |  | SD |
| 108 | 101 |  | 5·6 |

*Table 5. Results of a survey on the number of automobiles, by State, that switched from lead-free to leaded gasoline (0·05 g Pb/gal.).*

| State | Percentage |
|---|---|
| Tennessee | 9·7 |
| Delaware | 1·9 |
| Minnesota | 7·2 |
| Vermont I | 15·2 |
| New Jersey | 1·6 |
| Texas | 10·4 |
| Vermont II | 29·1 |
| Virginia | 6·0 |
| Arizona | 2·2 |
| Average | 9·3 |

*Table 6. The effect of time, temperature and type of container on the lead content of gasoline samples.*

| Container type |  | 1 Ref. | 1 Amb. | 2 Ref. | 2 Amb. | 4 Ref. | 4 Amb. | 23 Ref. | 23 Amb. |
|---|---|---|---|---|---|---|---|---|---|
| Polyethylene | $\bar{x}$ | 0·057 | 0·059 | 0·058 | 0·065 | 0·061 | 0·076 | 0·100 | 0·156 |
|  | $3\sigma$ | 0·009 | 0·002 | 0·012 | 0·007 | 0·006 | 0·007 | 0·007 | 0·003 |
| Soldered seam | $\bar{x}$ | 0·051 | 0·063 | 0·061 | 0·063 | 0·058 | 0·069 | 0·087 | 0·093 |
|  | $3\sigma$ | 0·010 | 0·002 | 0·008 | 0·011 | 0·012 | 0·005 | 0·009 | 0·017 |
| Pressed seam | $\bar{x}$ | 0·054 | 0·059 | 0·057 | 0·065 | 0·053 | 0·060 | 0·073 | 0·078 |
|  | $3\sigma$ | 0·007 | 0·002 | 0·006 | 0·009 | 0·003 | 0·005 | 0·005 | 0·020 |
| Pressed seam with insert | $\bar{x}$ | 0·060 | 0·061 | 0·060 | 0·063 | 0·055 | 0·065 | 0·092 | 0·084 |
|  | $3\sigma$ | 0·002 | 0·006 | 0·007 | 0·006 | 0·003 | 0·006 | 0·022 | 0·002 |
| Glass with LPE liner | $\bar{x}$ | 0·059 | 0·058 | 0·058 | 0·056 | 0·058 | 0·059 | 0·061 | 0·061 |
|  | $3\sigma$ | 0·003 | 0·000 | 0·012 | 0·018 | 0·006 | 0·012 | 0·002 | 0·002 |
| Glass with Teflon liner | $\bar{x}$ | 0·059 | 0·057 | 0·057 | 0·058 | 0·058 | 0·057 | 0·057 | 0·057 |
|  | $3\sigma$ | 0·006 | 0·006 | 0·012 | 0·012 | 0·003 | 0·003 | 0·003 | 0·004 |

Time (weeks): 1, 2, 4, 23.

Initial lead content was 0·056 ± 0·014 g Pb/gal. for all but glass containers which were 0·058 ± 0·006 g Pb/gal.
Where all values are in g Pb/gal.; Ref. = refrigerated; Amb. = ambient.

### *Sample storage study*

Polyethylene containers, tin containers with a lead-solder seam, tin containers with a pressed seam, tin containers with pressed seams with a stainless steel insert, glass containers with linear polyethylene liners, and glass containers with Teflon liners were evaluated and the effect on the lead content of the composite gasoline samples measured. Containers were stored at 4°C and ambient temperature and were analysed periodically. Table 6 summarizes the results of this investigation. The values reported are an average of the analysis of three individual samples. The initial lead content of the composition gasoline samples were 0·056 ± 0·014 g Pb/gal. for all the containers except glass, and 0·058 ± 0·006 g Pb/gal. for the glass containers. Initial values are based on seven analyses. At the end of one and two weeks, the lead concentrations remained within the initial value ranges. The polyethylene sample container stored at ambient temperatures start to show a concentrating effect at the fourth week. The worst case is the polyethylene container stored at ambient temperatures, while the best is the glass container. The concentrating effect is caused by the loss of the lighter weight gasoline fractions. Emission of these vapours is readily detected by the odour emitted from the polyethylene containers.

The results of this study illustrate that the storage time and the types of containers used need to be specified in the standard method. This is important to ensure legally defensible analyses. In consideration of the results presented in table 6, all samples should be collected in glass containers and analysed within four weeks. Within this time-frame, little difference is observed between storage at 4°C and ambient temperatures.

### References

1. American Society for Testing Materials (ASTM), *Method D 2337*, Part 17 (1973). (ASTM, 1916 Race Street, Philadelphia, Pennsylvania, USA.)
2. *Federal Register*, 15449 (28 July 1974).
3. Kashiki, M., Yamazoe, S. and Ohima, S., *Analytica Chimica Acta*, **53** (1971), 95.
4. Lukasiewiez, R. J., Berens, P. H. and Buell, B. E., *Analytical Chemistry*, **47** (1975), 1045.
5. Heistand, R. N. and Shaner, W. C., Jr., *Atomic Absorption Newsletter*, **13** (1974), 65.

